# Young Adults Coping with Sibling Loss: Risk and Protective Factors for Substance Use Disorder

**DOI:** 10.1007/s40653-025-00758-2

**Published:** 2025-09-03

**Authors:** Bat-Galim Shaer, Zehavit Gross, Yaniv Efrati

**Affiliations:** Faculty of Education, Bar-Ilan University, Ramat Gan, Israel

**Keywords:** Substance use disorder, Coping with sibling loss, Psychopathology, Bereavement guilt, Young adults

## Abstract

**Purpose:**

Although significant progress has been made in understanding substance use disorder (SUD), little research has specifically examined risk and protective factors among young adults coping with sibling loss. While some bereaved individuals develop SUD, others successfully implement protective strategies.

**Methods:**

The current study (*N* = 490; 260 bereaved, 230 non-bereaved) explored the associations of risk and protective factors with SUD, grouped into three categories: psychopathology (depression, anxiety, stress), grief-related aspects (personal growth, guilt, self-blame, regret, inadequacy, self-hatred, self-reassurance, stigma toward loss), and interpersonal relationships (quality of parental communication).

**Results:**

Higher-quality communication with parents was associated with a lower likelihood of developing SUD. Additionally, the relationships between depression, stress, and SUD were significantly moderated by sibling loss. Specifically, among bereaved young adults, increased guilt (mediated by anxiety and depression) heightened SUD risk, whereas increased feelings of inadequacy and stigma toward loss were associated with reduced risk.

**Conclusions:**

These findings underscore the importance of targeted support and interventions for young adults experiencing sibling loss, highlighting pathways that can reduce vulnerability to SUD.

Sibling bereavement is a profoundly distressing childhood experience that can function as both a traumatic event and an adverse developmental context, potentially leading to long-term physical and psychological consequences, including increased risk for substance use (Bolton et al., 2016; Burns et al., 2020; Fletcher et al., 2013). Despite its significance, research on sibling bereavement remains sparse (D’Alton et al., 2022; Halliwell & Franken, 2016; Kendler et al., 2023; Wagner et al., 2022). While not universal, young adulthood is often associated with increased engagement in behaviors such as alcohol consumption (Hammerton et al., 2023), cigarette smoking (Romm et al., 2023), use of electronic cigarettes (Thoonen & Jongenelis, 2023), and cannabis use (Albaugh et al., 2023), although recent evidence suggests shifts in these patterns among younger generations (Ball et al., 2024). However, bereaved siblings may be at greater risk for engaging in such behaviors due to the psychological impact of their loss. Grief-related emotional dysregulation, increased vulnerability to stress, and unmet attachment needs may drive some individuals toward substance use as a maladaptive coping strategy (Bolton et al., 2016; Burns et al., 2020). When used persistently to manage emotional pain, these behaviors can interfere with social and occupational functioning and contribute to heightened distress and negative affect (Volkow & Blanco, 2023). The goal of preventive science in the field of addictive behaviors is to prevent or moderate problematic behavior (Hunt et al., 2024) by identifying risk and protective factors early and implementing targeted interventions before maladaptive patterns become entrenched (O’Connell et al., 2009). Given the chronic and relapsing nature of many addictions, preventive science plays a critical role in reducing the personal and societal burden of addiction through early, cost-effective strategies that promote resilience and healthy development (Stanis & Andersen, 2014; Singh et al., 2022). In the current study, we examined protective and risk factors for young adults coping with sibling loss, with the aim of detecting young adults at risk of developing substance use disorder (SUD).

To guide this inquiry, we draw on The Multidimensional Grief Theory (MGT) (Kaplow et al., 2012, 2023), a developmentally-informed framework that categorizes grief reactions into three dimensions—Separation Distress, Existential/Identity Distress, and Circumstance-Related Distress—each of which can include both adaptive and maladaptive processes (Kaplow et al., 2013; Layne et al., 2017). This theory identifies three core dimensions of grief reactions—Separation Distress, Existential/Identity Distress, and Circumstance-Related Distress—each of which can involve both negative and positive adjustment processes (Kaplow et al., 2013; Layne et al., 2017). While it has been instrumental in guiding assessments and interventions that address grief-related psychopathology, such as depression and PTSD (Hill et al., 2019), MGT also acknowledges that adaptive outcomes can emerge alongside or even through the grieving process. In this regard, grief is understood as a multifaceted and developmentally-informed process, shaped by personal, relational, and socio-environmental factors (Kaplow et al., 2018).

To further contextualize this adaptive potential, Relational Developmental Systems (RDS) metatheory (Lerner et al., 2018; Overton, 2015) offers a complementary perspective. RDS emphasizes that development is dynamic and transactional, arising from reciprocal interactions between the individual and their proximal (e.g., family, school) and distal (e.g., cultural) ecologies. In the context of bereavement, RDS posits that youth have the potential for positive adaptation, especially when their developmental needs are met by contextual assets—such as supportive caregiving, effective communication, and social connection (Kentor & Kaplow, 2020). When applied to sibling loss, these frameworks collectively allow for a more nuanced understanding of how risk factors (e.g., guilt, self-criticism) and protective factors (e.g., parental communication, meaning-making, personal growth) co-occur and interact over time. This integrated approach aligns with the current study’s focus on identifying both vulnerability and resilience among bereaved youth in relation to substance use outcomes (Alvis et al., 2023; D’Alton et al., 2022).

Research has extensively explored various risk factors associated with the development of SUD (Merikangas, & McClair, 2012). With regard specifically to sibling loss, bereavement-related guilt (i.e., guilt feelings experienced by bereaved individuals), self-blame, regret, and additional stress-inducing experiences related to the loss—such as witnessing the sibling’s death or facing family disruption—are widely recognized as risk factors for several psychiatric disorders, including SUD (Masferrer et al., 2017).From the earliest research on bereavement, the following have consistently been identified as among the most frequent reactions to losing a loved one: guilt, self-blame, and regret (Lindemann, 1944; Keser et al., 2022). Stroebe et al. (2014) described guilt as a complex construct with both cognitive and emotional dimensions, comprising various components such as self-blame and regret. Self-blame in this context refers to the belief that one is personally responsible for the death of another (Davis et al., 1996; Weinberg, 1994). Regret involves the negative emotional experience accompanied by the belief that different actions could have improved the outcome related to the deceased’s death or the events surrounding it (Stroebe et al., 2014). Li et al. (2014) performed a comprehensive update of both quantitative and qualitative studies examining guilt associated with bereavement. Their findings indicated that guilt was connected to poorer physical health, traumatic responses, and symptoms of grief. Further, longitudinal research has shown that self-blame and guilt can predict the severity of grief symptoms that follow a loss (Li et al., 2019; LeBlanc et al., 2020).

In the literature on bereavement, psychopathology—including depression, anxiety, and stress—has been identified as a primary risk factor for the development of maladaptive coping behaviors and mental health disorders, including SUD, among individuals experiencing the loss of a sibling during young adulthood (Herberman et al., 2013). Previous longitudinal studies provide compelling evidence of the psychological impact of bereavement in young adulthood, supporting the rationale for examining risk factors such as psychopathology and substance use. For example, a prospective study of 2,230 Dutch young adults found that those who experienced the loss of a parent or sibling exhibited significantly more internalizing problems within two years post-bereavement compared to their non-bereaved peers, with 22% developing new internalizing symptoms versus 5.5% in the control group (Stikkelbroek et al., 2016). Similarly, Denckla et al. (2023) demonstrated that bereavement (due to the loss of a parent, sibling, or close friend) was associated with elevated emotional and behavioral symptoms across several domains. These findings underscore the heightened vulnerability of bereaved young adults to psychological distress, reinforcing the need to investigate pathways that may contribute to or mitigate maladaptive outcomes, including SUDs.

Another important risk factor is internalized stigma, in which individuals view their grief responses as socially unacceptable or psychologically problematic— a perception that has been linked to increased risk for SUD in the context of bereavement (Kulesza et al., 2017). This form of stigma originates from an individual’s own evaluations of their psychological distress or psychiatric conditions, and their beliefs about the implications of such experiences (Sprang, & McNeil, 2018). According to Relational Frame Theory (RFT; Hayes et al., 2001), a modern behavior-analytic framework that explains complex human behaviors, such self-stigma can lead individuals to embrace negative stereotypes or labels about their condition instead of recognizing their evolving mental states. This critical and interconnected response to internal experiences, such as negative self-talk and internalized shame, may intensify self-stigma, leading to prolonged psychological distress and addictive behaviors, as indicated in recent studies (Krafft et al., 2018; Martin et al., 2022; Efrati, 2023).

Closely tied to stigma is self-criticism, which has been linked to a heightened risk of SUD, particularly among those who experience profound feelings of unworthiness or failure following loss (Caparrós & Masferrer, 2021). Individuals with this trait often engage in rigorous self-assessment, accompanied by a fear of disapproval and a deep-seated need for others’ approval and acceptance. The experience of sibling bereavement can amplify self-critical behaviors, particularly if the individual struggles with bereavement-related guilt, self-blame, or regret. Such feelings may lead to negative self-perceptions, such as a sense of inferiority or self-loathing, which can contribute to various psychological issues (Gilbert et al., 2004). Self-criticism has been strongly linked to psychopathology (Blatt et al., 1982; McIntyre et al., 2018), and within the framework of the Transactional Model of Stress and Coping (Lazarus & Folkman, 1984), it is considered a key factor that may influence the progression from stress to psychopathology (Kotera et al., 2022).

Although many young adults are exposed to significant risk factors—such as sibling loss, self-criticism, and bereavement-related guilt—not all go on to develop SUD, highlighting the potential role of protective factors that buffer against such outcomes (Nawi et al., 2021; Stone et al., 2012). One key protective factor identified in the literature is effective communication with parents, which has been shown to mitigate the likelihood of engaging in substance use (Leadbeater et al., 2022; Middleton et al., 2017; Stormshak et al., 2019). For bereaved young adults in particular, parental support and open communication have been found to play a critical role in the coping process, while parental distress may contribute to the child’s psychological vulnerability (D’Alton et al., 2022). Drawing on the family systems perspective (Erel & Burman, 1995), parent-adolescent communication is conceptualized as a dynamic and reciprocal process in which adolescents actively contribute by disclosing information about their experiences and emotions (Stattin & Kerr, 2000). However, discrepancies often arise between parents’ and adolescents’ perceptions of these interactions, with adolescents frequently evaluating parental involvement more negatively (Hou et al., 2020; Janssens et al., 2015; Zhou et al., 2023). Such perceptual gaps may reflect normative developmental processes (Phinney et al., 2005) or deeper relational strain (Guion et al., 2009). Nevertheless, studies consistently show that open, supportive communication strengthens the parent-child bond and serves as a protective factor against various maladaptive outcomes—including delinquent behavior (Kapetanovic et al., 2019) and behavioral addictions (Efrati, 2023)—and may similarly protect against the development of SUD following sibling bereavement.

## The Current Study

It is well-recognized that the grief experienced after the loss of a sibling can lead to psychological and functional impairments, including an increased risk to develop a SUD. The main goal of the current study was to identify risk and protective factors associated with the likelihood of SUD among young adults who have experienced the loss of a sibling. To this end, we focused on variables supported by prior research and theory in the domains of psychopathology, grief processing, and family relationships. Specifically, we examined three overarching categories: psychopathology (depression, anxiety, stress), grief-related factors (personal growth, guilt, self-blame, regret, feelings of inadequacy, self-hatred, self-reassurance, and stigma related to loss), and interpersonal relationships (specifically, the quality of communication with parents). Gaining a deeper understanding of these risk and protective factors may enhance the support provided to young adults coping with the loss of a sibling and potentially reduce maladaptive coping mechanisms. Based on previous literature, we expected that psychological distress and the quality of parent–young adult communication would interact with bereavement status in predicting SUD. We also anticipated that, within the bereaved group, individual differences in grief responses, self-related processes, and interpersonal factors would be associated with the likelihood of SUD. This integrative approach aims to elucidate how specific risk, and protective factors jointly shape vulnerability to SUD in bereaved young adults, offering valuable insight into avenues for prevention and targeted intervention.

## Method

### Participants

The study population comprised 490 Jewish Israeli adolescents and young adults, including individuals who had lost a sibling (*n* = 260; M_age = 17.0, SD = 3.5) and those from the general community with no such loss (*n* = 230; M_age = 16.8, SD = 2.5). Participants ranged in age from 14 to 25 years. Most participants (96%) were native Israelis, and 92% reported Hebrew as their first language. Additional demographic details are presented in Table 1.

**Table 1:** Young adults (*n* = 490) means, standard deviations, and percentage frequencies

Coping with Sibling Loss							
		Yes (*n* = 260) No (*N* = 230)			Yes (*n* = 260) No (*N* = 230)		
		%	M	SD	%	M	SD
Gender	Male	41%			43%		
	Female	58%			57%		
Age	14–25		17.00	3.5		16.8	2.5
Grade	7th to 9th	12.8%			1.3%		
	10th and 12th	47.2%			77.3%		
	Post-school	40%			20.6%		
Country of birth	Israel	95%			97%		
	Other	5%			3%		
Socioeconomic status	Below average	4.6%			3.5%		
	Average	65%			67%		
	Above average	30%			29%		
Language	Hebrew	92%			92%		
	Other	8%			8%		
The type of bereavement	Accident	6.2%					
	Military Service	12%					
	Terrorist attack	26%					
	Disease	38%					
	Other	19%					
The range of years since the loss	0–3	36%					
	4–8	29%					
	9–15	22%					
	15<	12%					
Did you use any professional therapy to cope with the loss?	Yes	48%					
	No	52%					

### Measures

All grief-related instruments were translated into Hebrew using the back-translation method to ensure conceptual equivalence. Participants responded using Likert-type scales, and internal consistency for each measure was evaluated using Cronbach’s alpha and McDonald’s omega. A quantitative design was chosen to systematically test the relationships among key risk and protective factors related to SUD. This approach allowed for the examination of interaction effects (e.g., bereavement status) and supported generalizable, data-driven conclusions to inform intervention efforts.

**Sociodemographic variables**. Participants provided information regarding their age (14–18 years), sex assigned at birth (male or female), immigration status (native Israeli or immigrant), and socioeconomic status (SES). SES was assessed using a single-item measure with four response options: very good, good, bad, and very bad. Because very few participants selected *bad* or *very bad*, these two categories were combined to ensure sufficient sample size for statistical analysis and to improve the stability and interpretability of the findings. Accordingly, SES was recoded into three levels: (1) very good, (2) good, and (3) bad/very bad.

Given the multifaceted nature of grief, multiple validated scales were employed to capture distinct dimensions of the bereavement experience (Bonanno, & Kaltman, 2001), including self-blame, guilt, personal growth, and internalized stigma.

**Self-Blame and Regret in Coping with Loss**. Self-blame and regret were assessed using a 10-item scale adapted from the Tübingen Bereavement Symptoms Questionnaire (Stroebe et al., 2014). The scale includes two subscales—self-blame (e.g., “I often wish I could have died instead of him”) and regret (e.g., “If I could be with him one more time, I’d do things a lot differently”)—each rated on a 5-point Likert scale. Cronbach’s alpha and McDonald’s omega values were 0.64 (ω = 0.75) for self-blame and 0.89 (ω = 0.92) for regret.

**Bereavement Guilt**. Bereavement guilt was measured using the 14-item Bereavement Guilt Scale (BGS; Li et al., 2017), comprising five domains: responsibility, hurting the deceased, survivor guilt, indebtedness, and general guilt. A total score was computed, with higher scores indicating greater guilt. Internal consistency was α = 0.90 (ω = 0.92).

**Grief and Personal Growth**. Grief and personal growth were assessed with the 21-item Hogan Inventory of Bereavement Short Form for Children and Adolescents (HIB-SF-CA; Hogan et al., 2021). The scale includes 10 items on grief (e.g., “I do not sleep well at night”) and 11 on personal growth (e.g., “I am more aware of others’ feelings”), rated on a 5-point Likert scale. Internal consistency for grief was α = 0.82 (ω = 0.87) and for personal growth α = 0.91 (ω = 0.93).

**Internalized Stigma of Bereavement**. Self-stigma due to bereavement was assessed using a 10-item scale based on Ritsher et al. (2003) and Boyd et al. (2014). Responses were rated on a 4-point Likert scale (e.g., “People discount the things I say because I am grieving”). Internal consistency was α = 0.72 (ω = 0.78).

**Parent-Adolescent Communication**. The Parent-Adolescent Communication Scale (PACS; Barnes & Olson, 1985) was used to measure communication quality, comprising 20 items across two subscales: open communication (e.g., “My parents answer my questions honestly”) and communication problems (e.g., “My parents tend to say things that would be better left unsaid”). The PACS has been extensively used in Hebrew (e.g., Efrati et al., 2024; Efrati, 2024) and has been found to be a valid and reliable measure. Responses were rated on a 5-point Likert scale. Internal consistency was α = 0.91 (ω = 0.93).

**Depression**, **Anxiety**, **and Stress**. Depression, anxiety, and stress were measured using the 21-item Depression, Anxiety, and Stress Scale (DASS-21; Lovibond & Lovibond, 1995). The DASS has also been extensively used in Hebrew (e.g., Efrati, 2023) and found to be valid and reliable. Items were rated on a 4-point scale, assessing symptoms experienced in the past week (e.g., “I felt that I had nothing to look forward to”). Internal consistency was α = 0.91 (ω = 0.93) for depression, α = 0.90 (ω = 0.93) for anxiety, and α = 0.90 (ω = 0.93) for stress.

**Self-Criticism and Self-Reassurance**. These constructs were assessed using the 22-item Forms of Self-Criticizing and Self-Reassuring Scale (FSCRS; Gilbert et al., 2004). The FSCRS has also been extensively used in Hebrew (e.g., Shahar, 2015) and found to be valid and reliable. The scale includes subscales for inadequate self (e.g., “There’s a part of me that feels I am not good enough”), hated self (e.g., “I want to hurt or injure myself”), and self-reassurance (e.g., “I am able to remind myself of positive things about myself”), rated on a 5-point scale. Internal consistencies were α = 0.90 (ω = 0.92), α = 0.81 (ω = 0.84), and α = 0.86 (ω = 0.88).

**Substance Use Disorder (SUD)**. SUD was evaluated using four standardized instruments: the Alcohol Use Disorder Identification Test (AUDIT-10; Saunders et al., 1993), the Fagerström Test for Nicotine Dependence (Heatherton et al., 1991), the Penn State Electronic Cigarette Index (Foulds et al., 2015), and the Cannabis Use Disorder Identification Test-Revised (CUDIT-R; Adamson et al., 2010). Participants meeting the clinical threshold for any of the tools were coded as 1 (SUD present); others were coded 0. This dichotomous scoring approach was applied due to low base rates for individual SUD diagnoses.

### Procedure

The study was introduced as research on young adults coping with sibling loss. Participants were recruited from across Israel (eastern, central, southern, and northern regions), including both bereaved and non-bereaved individuals. Bereaved participants were recruited through support organizations (e.g., “One Family”) and in-person outreach at related events. Non-bereaved participants were recruited in person and matched to the bereaved group on age, gender, and socioeconomic status (SES). Surveys were administered online via Qualtrics and could be completed on a computer or smartphone. Participation was voluntary and anonymous. For individuals over 18, written informed consent was obtained. For those under 18, parental consent was secured via email or phone, followed by adolescent assent. All participants were asked to complete the survey independently in a quiet setting. The average completion time was approximately 24 min. No compensation was provided. All measures were administered in Hebrew, the native language of most participants. The order of questionnaires was randomized. An online debriefing followed the survey. The study was approved by the university’s Institutional Review Board (IRB).

### Data Analysis

Overall, 17.10% of the data were missing. To examine the pattern of missingness, we employed Jamshidian and Jalal’s non-parametric Missing Completely At Random (MCAR) test. The analysis indicated that the data were MCAR, *Hawkins’ χ*^*2*^_(18)_ = 69.67, *p* = 5.13^^−8^, *Anderson-Darling T* = 8.69, *p* = .32. Accordingly, we handled the missing data using multiple imputations (MI; Rubin, 2009) with the *mice* R package. Specifically, using MI, we constructed 18 complete datasets (equivalent to the percentage of missing data), the analyses were conducted on each dataset and then pooled.

**Fig. 1:**
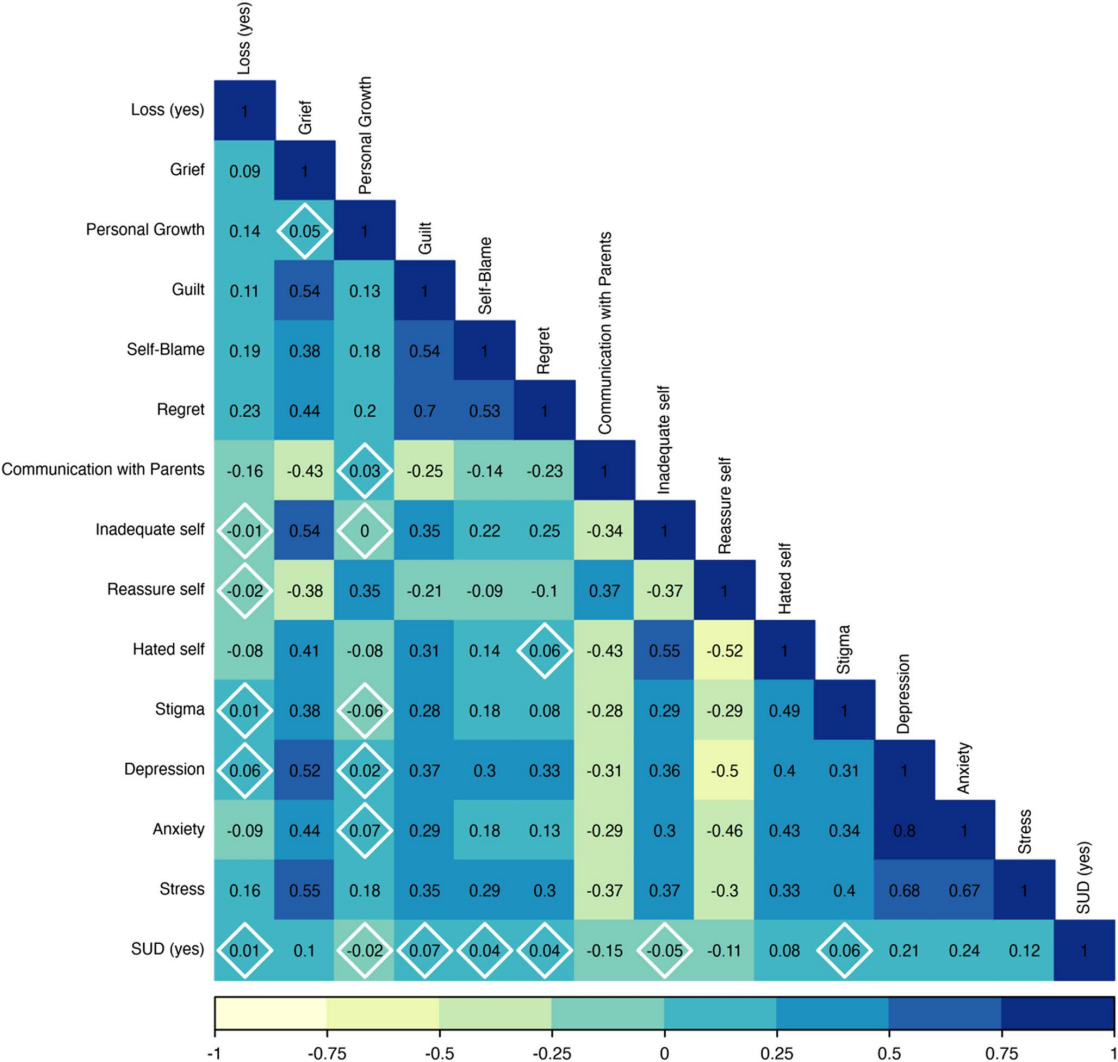
The pattern of associations between study measures. SUD = Substance Use Disorder. Values within a white diamond are not statistically significant. All other values are *p* < .05

We first examined bivariate associations between study variables using Pearson correlations (see Fig. 1). To identify potential influential data points that could bias regression results, we applied the *influencePlot* function from the *car* R package, which revealed five such points. Consequently, we conducted robust regression analyses using the *robustbase* R package. To test moderation effects, we employed a hierarchical robust logistic regression. In Step 1, we entered death of a sibling (yes/no), quality of communication with parents, and psychopathology indicators (depression, anxiety, stress). In Step 2, we added two-way interactions among these variables. Simple slopes analyses were conducted for significant interactions using the *interactions* R package. Direct effects were interpreted from Step 1. To appraise the stability of the effects, we added a third step in which we controlled for the effects of four covariates: type of bereavement (accident, military, terror attack, illness, and other), time since the bereavement, gender, and age.

Next, we examined predictors of SUD among bereaved participants. A separate hierarchical robust logistic regression was conducted. Step 1 included grief, personal growth, guilt, self-blame, regret, quality of communication with parents, self-related constructs (inadequate, reassured, hated self), and perceived stigma. Step 2 added psychopathology indices to assess the incremental predictive value of these variables. Significant effects were visualized using the *effects* R package. For predictors losing significance after controlling for psychopathology, we tested potential mediation via structural equation modeling (SEM) using the *lavaan* package with Diagonally Weighted Least Squares estimation and bias-corrected bootstrap errors. As in the previous analysis, to appraise the stability of the effects, we added a third step in which we controlled for the effects of four covariates: type of bereavement (accident, military, terror attack, illness, and other), time since the bereavement, gender, and age.

## Results

### Associations between Study Variables and Direct Effects on SUD Likelihood

Figure 1 presents the pattern of associations between study measures. Results are presented in Table 2. The robust logistic regression revealed that higher quality communication with parents was associated with a lower likelihood of SUD. Specifically, a one-point increase in the quality of communication with parents was related to a 28% decrease in the probability of SUD. In addition, the greater the severity of anxiety and the lower the stress, the higher the likelihood of having a substance use disorder; a one-point increase in anxiety was associated with an increase of 92% in the probability of SUD, whereas a one-point increase in stress was associated with a decrease of 34% in the likelihood of SUD. These direct effects accounted for 7.9% of the variance in the probability of having SUD.

**Table 2:** Robust logistic regression coefficients for predicting the likelihood of substance use disorder by coping with a loss, quality of communication with parents, psychopathology and their interactions

Predictors	SUD (Step 1)			SUD (Step 2)			SUD (Step 3)		
	Odds Ratios	CI	*p*	Odds Ratios	CI	*p*	Odds Ratios	CI	*p*
(Intercept)	0.43	0.32–0.57	**< 0.001**	0.42	0.31–0.57	**< 0.001**	0.48	0.07–3.18	0.445
Coping with a loss (no)	0.83	0.54–1.30	0.418	0.87	0.55–1.38	0.551	0.60	0.32–1.14	0.116
Quality of communication with parents	0.72	0.53–0.97	**0.033**	0.75	0.48–1.18	0.213	0.81	0.50–1.30	0.375
Depression	1.16	0.79–1.71	0.454	1.79	1.06–3.00	**0.029**	1.62	0.93–2.82	0.089
Anxiety	1.92	1.29–2.87	**0.001**	1.89	1.09–3.27	**0.023**	2.20	1.22–3.97	**0.009**
Stress	0.66	0.47–0.93	**0.016**	0.44	0.27–0.74	**0.002**	0.42	0.25–0.72	**0.001**
Coping x Quality of communication				0.89	0.48–1.67	0.724	0.81	0.42–1.56	0.532
Coping x Depression				0.38	0.17–0.85	**0.019**	0.39	0.17–0.91	**0.030**
Coping x Anxiety				1.21	0.53–2.77	0.648	1.19	0.50–2.85	0.692
Coping x Stress				2.15	1.07–4.32	**0.032**	2.10	1.02–4.31	**0.044**
Accident vs. Military							0.96	0.23–3.96	0.957
Accident vs. Terror Attack							2.32	0.65–8.31	0.195
Accident vs. Illness							1.71	0.50–5.83	0.393
Accident vs. Other							2.26	0.68–7.50	0.181
Years Since Bereavement							0.81	0.62–1.06	0.117
Gender							0.63	0.40–0.98	**0.042**
Age							1.03	0.95–1.11	0.464
Observations	490			490			490		
R^2^ Tjur	0.079			0.100			0.121		

Note. SUD = substance use disorder; CI = 95% confidence interval for the odds ratios

### Interaction Effects: Moderating Role of Stress and Depression by Sibling Loss

**Fig. 2:**
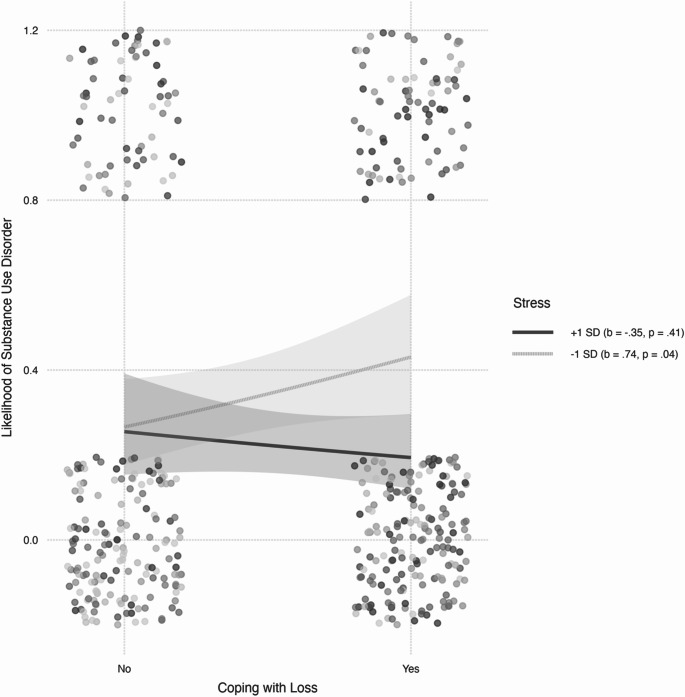
The association between coping with the loss of a sibling and the likelihood of substance abuse disorder as a function of participants’ stress

**Fig. 3:**
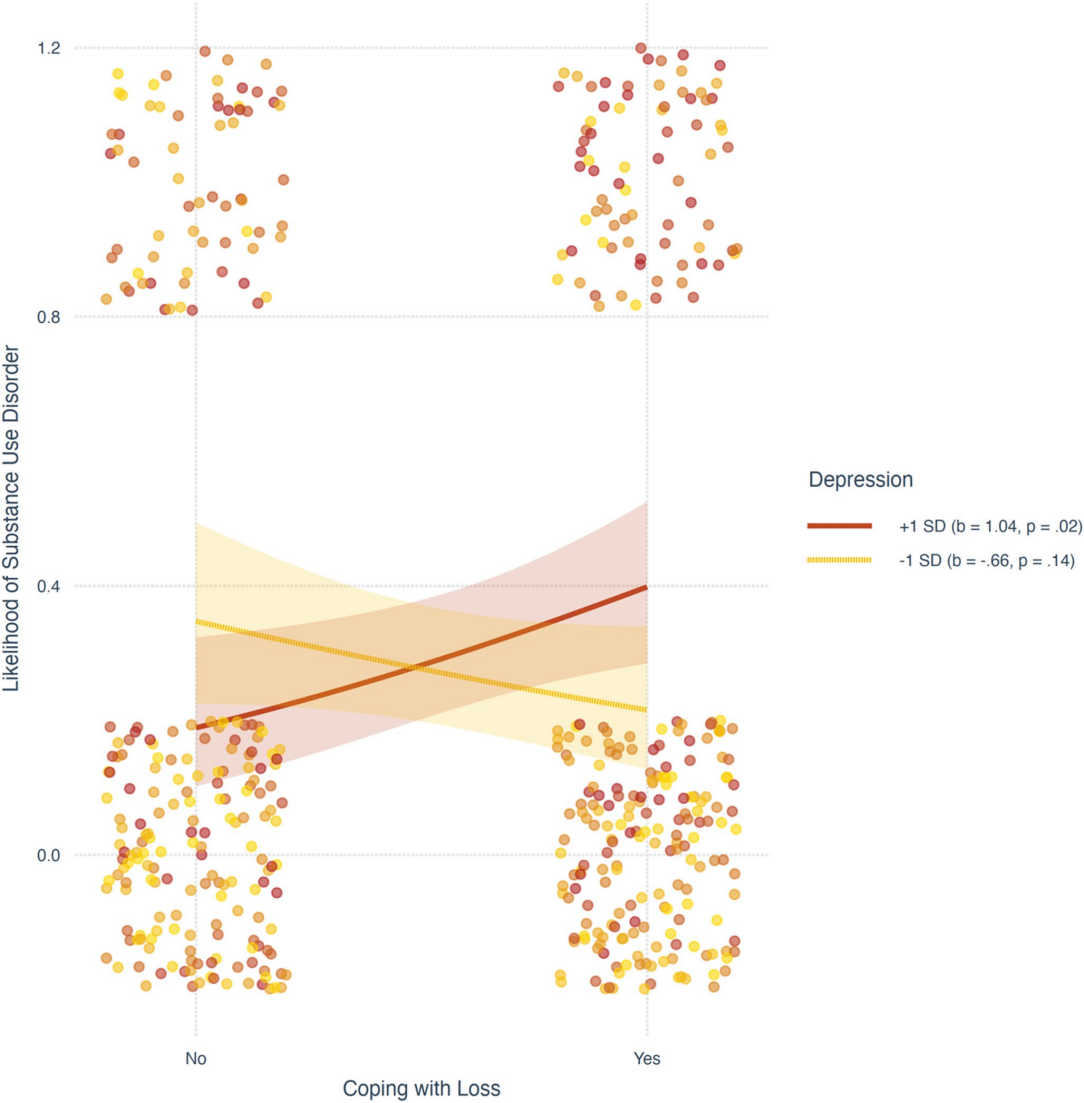
The association between coping with the loss of a sibling and the likelihood of substance abuse disorder as a function of participants’ depression

The model also revealed that the association between depression, stress, and the likelihood of having SUD was significantly moderated by coping with the loss of a sibling (i.e., significant interactions). Simple main effects tests indicated that among participants high in stress (one standard deviation above the sample mean), the likelihood of having SUD was similar for participants who lost a sibling and those who did not (*b* = -0.35, *SE* = 0.43, *t* = -0.82, *p* = .412). Conversely, among participants low in stress (one deviation below the sample mean), the likelihood of having SUD was significantly higher for participants who lost a sibling than those who did not (*b* = 0.74, *SE* = 0.36, *t* = 2.05, *p* = .041; see Fig. 2). Regarding depression, the tests indicated that among participants low in depression (one standard deviation below the sample mean), the likelihood of having SUD was similar for participants who lost a sibling and those who did not (*b* = -0.66, *SE* = 0.44, *t* = -1.49, *p* = .137). Conversely, among participants high in depression (one deviation above the sample mean), the likelihood of having SUD was significantly higher for participants who lost a sibling than those who did not (*b* = 1.05, *SE* = 0.45, *t* = 2.33, *p* = .020; see Fig. 3). The interactions added 2.1% to the explained variance of SUD. As noted in Table 2, the addition of type of bereavement (accident, military, terror attack, illness, and other), time since the bereavement, gender, and age as covariates did not change the pattern of associations.

### Predictors of SUD Within the Bereaved Group

**Fig. 4:**
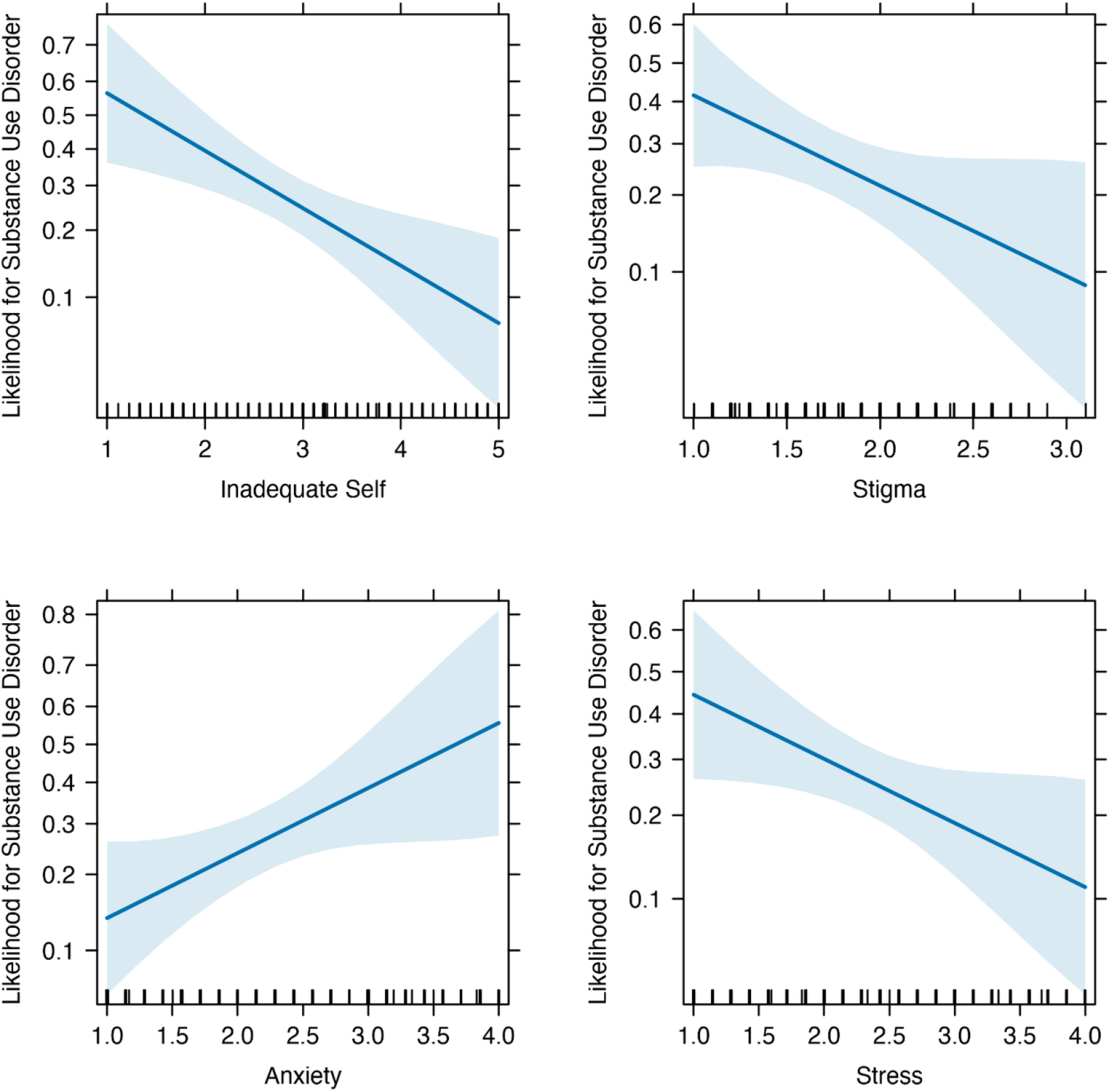
The association between anxiety, stress, stigma, and a sense of inadequate self and the likelihood of substance abuse disorder only among individuals who lost a sibling (taken from Step 2 of the analysis)

Results are presented in Table 3. The robust logistic regression revealed three significant effects in Step 2 that significantly predicted the probability of having SUD among participants who lost a sibling. Specifically, we found that for a one-point increase in the sense of guilt, the likelihood of having SUD increased by 86%. Conversely, a one-point increase in the sense of inadequate self and/or stigma toward loss was associated with a decrease of 54% and 63%, respectively, in the probability of having SUD. After the inclusion of the psychopathology indices – depression, anxiety, and stress – in the second step of the model, the association between inadequate self, stigma toward loss, and SUD remains significant, whereas the association between guilt and SUD became nonsignificant. Accordingly, we continued with examining whether depression, anxiety, and stress mediated the effect of guilt on SUD. The significant predictions of the complete model (i.e., Step 2) are presented in Fig. 4. As noted in Table 3, the addition of the type of bereavement (accident, military, terror attack, illness, and other), time since the bereavement, gender, and age as covariates incurred one change in the pattern of results: the significant effect of stress on the likelihood of substance use disorder (*p* = .036) became only marginally significant (*p* = .066).

**Table 3:** Robust logistic regression coefficients for predicting the likelihood of substance use disorder only among participants who lost a sibling

Predictors	SUD (Step 1)			SUD (Step 2)			SUD (Step 3)		
	Odds Ratios	CI	*p*	Odds Ratios	CI	*p*	Odds Ratios	CI	*p*
(Intercept)	9.87	0.44–221.06	0.149	0.90	0.03–30.40	0.952	2.70	0.03–229.05	0.662
Grief	1.56	0.85–2.85	0.153	1.30	0.67–2.51	0.437	1.34	0.65–2.74	0.430
Personal Growth	1.07	0.74–1.57	0.714	1.02	0.67–1.54	0.935	1.02	0.65–1.59	0.944
Guilt	1.86	1.00–3.43	**0.049**	1.67	0.88–3.17	0.119	1.77	0.92–3.42	0.088
Self-Blame	1.24	0.75–2.03	0.401	1.29	0.78–2.14	0.321	1.26	0.75–2.14	0.384
Regret	0.86	0.59–1.27	0.457	0.84	0.55–1.27	0.409	0.87	0.56–1.36	0.545
Quality of communication with parents	0.79	0.48–1.29	0.347	0.80	0.47–1.37	0.416	0.87	0.49–1.54	0.633
Inadequate self	0.46	0.30–0.70	**< 0.001**	0.50	0.32–0.77	**0.002**	0.46	0.29–0.74	**0.001**
Reassure self	0.66	0.43–1.02	0.064	1.03	0.61–1.72	0.919	0.99	0.57–1.73	0.985
Hated self	1.14	0.69–1.86	0.610	1.13	0.67–1.89	0.652	1.01	0.58–1.75	0.978
Stigma	0.37	0.16–0.87	**0.022**	0.39	0.15–0.97	**0.043**	0.37	0.14–0.97	**0.043**
Depression				1.77	0.94–3.35	0.079	1.47	0.75–2.89	0.267
Anxiety				2.00	1.06–3.77	**0.033**	2.52	1.25–5.08	**0.009**
Stress				0.54	0.30–0.96	**0.036**	0.57	0.31–1.04	0.066
Accident vs. Military							0.60	0.12–3.04	0.540
Accident vs. Terror Attack							1.90	0.45–7.98	0.380
Accident vs. Illness							1.31	0.34–5.02	0.691
Accident vs. Other							1.41	0.33–6.04	0.645
Years Since Bereavement							0.80	0.55–1.17	0.252
Gender							0.51	0.25–1.04	0.065
Age							1.00	0.90–1.12	0.965
Observations	260			260			260		
R^2^ Tjur	0.120			0.190			0.220		

### Mediation Analysis: Psychopathology as a Mechanism Linking Guilt To SUD

The SEM multi-mediation path model indicated that the effect of participants’ sense of guilt on the likelihood of having a SUD was significantly and independently mediated by elevated depression (95% bias-corrected confidence interval for the indirect effect [CI_BC_indirect_] = 0.06, 0.24) and anxiety (CI_BC_indirect_ = 0.05, 0.19) but not stress (CI_BC_indirect_ = − 0.06, 0.11; i.e., included 0, which refers to the likelihood of a null effect). Specifically, guilt was associated with greater severity of depression (*b* = 0.45, *SE* = 0.07, *t* = 6.70, *p* < .001) and anxiety (*b* = 0.33, *SE* = 0.07, *t* = 4.64, *p* < .001), which in turn significantly predicted the likelihood of having a SUD.

## Discussion

The aim of the current study was to examine the role of risk and protective factors in predicting SUD among adolescents and young adults who have experienced the loss of a sibling. While prior research has explored the emotional and psychological impact of sibling bereavement during childhood and adolescence (D’Alton et al., 2022), limited attention has been given to its potential link with SUD in later developmental stages (Bolton et al., 2016). This study seeks to address this gap by identifying specific factors that may increase vulnerability or provide resilience in the aftermath of such a loss.

In the current study, we found that the impact of sibling loss on the likelihood of developing a substance use disorder (SUD) was moderated by levels of stress and depression. Interestingly, high stress levels did not differentiate the risk of SUD between those who had or had not experienced sibling loss. However, individuals reporting *low stress* who had lost a sibling were significantly more likely to develop SUD. One possible explanation, as suggested in prior research, is that individuals with low perceived stress may not have developed sufficient coping mechanisms for handling acute emotional distress (Cohen & McKay, 2020). When confronted with a major life event such as sibling bereavement, these individuals might be more vulnerable to maladaptive coping strategies, including substance use.

In contrast, those experiencing *high levels of depression* showed a significantly increased risk of SUD when also facing sibling loss—consistent with literature linking depressive symptoms with substance use as a form of emotional self-regulation (Fahmy et al., 2024). The absence of a moderating effect of *high stress* may also be explained by stress adaptation models (Ellis, & Del Giudice, 2019) which suggest that individuals with chronically high stress levels might develop certain resilience mechanisms or become desensitized to additional stressors (Charney, 2004; ). Alternatively, the type and source of stress (e.g., acute vs. chronic, interpersonal vs. academic) may influence its interaction with bereavement and substance use, a distinction not captured in the current study. Future research should further differentiate types of stress and explore potential mediating factors—such as emotion regulation capacity or social support—that may explain these interaction effects more comprehensively.

Regarding young adults who experienced the loss of a sibling, we unexpectedly found that an increase in “inadequate self” and/or stigma toward loss was correlated with a decreased likelihood of SUD. Even after accounting for the psychopathological factors of depression, anxiety, and stress, the relationships between inadequate self, stigma toward loss, and SUD remained significant. One possible explanation is that self-critical or stigmatized individuals may be more likely to engage in internalized processing and adaptive help-seeking behavior, particularly in the context of bereavement. Prior research suggests that experiences of stigma and feelings of inadequacy may prompt individuals to seek external validation, meaning-making, or social affiliation (Corrigan et al., 2014). In grief contexts, this often takes the form of participation in bereavement support groups or online communities, where individuals can process their emotions, gain peer support, and reduce isolation (Robinson, & Pond, 2019). These structured coping environments may serve as protective factors against maladaptive coping strategies such as substance use.

From a developmental perspective, late adolescence and young adulthood are critical periods for identity formation and moral reasoning (Moshman, 2005; Killen & Dahl, 2021). Feelings of inadequacy or perceived societal disapproval may trigger increased self-monitoring, particularly among those with high emotional sensitivity (Gangestad & Snyder, 2000; Lin & Guo, 2024). This may foster prosocial coping, such as turning to family, religion, or community resources—thereby reducing the reliance on substances to manage grief-related distress.

Rather than serving as risk factors, inadequate self and perceived stigma may, in some contexts, paradoxically activate protective processes that promote psychological adaptation and inhibit substance use. Future research should investigate whether these effects are moderated by levels of social support, cultural norms around grief expression, and accessibility of coping resources.

Research on bereavement underscores the pivotal role of guilt as a key risk factor in the grieving process (Stroebe et al., 2014; LeBlanc et al., 2020). Although we found no direct link between guilt and SUD, symptoms of SUD were more likely to appear among siblings who had experienced bereavement when mediated by anxiety and depression. Guilt, often manifested as self-blame, regret, and internal conflict, can prompt individuals contending with a sibling’s loss and subsequent anxiety and depression to resort to SUD as a means of alleviating emotional pain. This notion supports the argument that cognitive processes affect behavior and might explain the mediating role of anxiety and depression. Guilt may lead to negative thoughts (“I could have done more”) that contribute to anxiety and depression, which then increase the likelihood of SUD as a way of coping with these painful thoughts and feelings (Wild et al., 2023). However, we did not find a direct influence of guilt on SUD. An explanation for this (lack of) finding may be found in LeBlanc et al. (2020), which suggested that the impact of guilt on psychopathology was moderated by the level of shame experienced, indicating a complex interplay among these emotions. Future research should further examine how guilt, shame, and psychopathology collectively influence the development of SUD.

Results also indicated that the better the quality of communication with the parents, the lower the likelihood of having an SUD. This finding is consistent with findings from multiple studies documenting negative correlations between parent-child relationships and SUD (Leadbeater et al., 2022; Middleton et al., 2017; Stormshak et al., 2019). For instance, longitudinal data from a community sample of 593 parents and their children in the USA, collected over six years, examined differences in young adult alcohol and marijuana use and the quality of parent-young adult relationships. The findings indicated that high-risk alcohol and marijuana use was associated with poorer parent-young adult relationship quality. Additionally, the initiation of marijuana use during young adulthood was linked to worse parent-young adult relationship quality (Stormshak et al., 2019).

Overall, our findings emphasize that bereavement does not uniformly lead to SUD among young adults who lost a sibling. Instead, the presence of specific emotional and psychological factors, particularly when combined with poor coping strategies, significantly influences the pathway to SUD. For those experiencing bereavement in the absence of these risk factors, substance use may not present itself as a coping mechanism, highlighting the importance of targeted interventions that address both emotional and psychological needs.

Although our main premises were supported, it is important to acknowledge the significant limitations of the current study. First, the cross-sectional design limits our understanding of the temporal sequence of risk factors and their mediation by psychopathology – a model that, despite strong theoretical and empirical backing, requires validation through longitudinal research to clarify causal directions. Second, the correlational nature of this study limits our ability to infer causation, necessitating caution when applying these findings in clinical settings. Lastly, the sample was confined to Israeli Jewish young adults and adolescents; thus, diverse ethnic and cultural groups should be assessed in future studies, to examine the universality of these results.

Despite these limitations, the findings of this study align with established research and have significant implications for prevention programs. We recommend interventions that enhance parental communication, as its inverse relationship with SUD prevalence highlights the need to strengthen family dynamics. Such programs could be crucial in reducing the risk of SUDs by fostering open and supportive communication between parents and bereaved young adults/adolescents. Additionally, tailored mental health interventions are critical for young adults/adolescents who are more vulnerable (i.e., those who manifest low levels of stress and high levels of depression and guilt after the loss of a sibling). The aim of such interventions should be to boost their resilience and help them develop effective coping strategies to manage grief and prevent SUD. Support groups and community resources could be particularly beneficial for those feeling a sense of inadequacy or experiencing loss-related stigma, as such groups could provide social support and alternative coping mechanisms, thus reducing the likelihood of SUD development. Finally, policymakers should consider these findings when developing comprehensive bereavement care policies that incorporate mental health support, acknowledging the profound impact that the loss of a sibling can have on young adults and adolescents.

## Data Availability

The datasets generated during and/or analysed during the current study are available from the corresponding author on reasonable request.
